# Does composite repair time affect repair protocol, immediate or delayed?

**DOI:** 10.1186/s12903-025-07509-7

**Published:** 2025-12-15

**Authors:** Murat Can Ersen, Nevin Cobanoglu

**Affiliations:** 1 Faculty of Dentistry Department of Restorative Dentistry, Bursa Uludag University, Bursa, Türkiye; 2 Faculty of Dentistry Department of Restorative Dentistry, Selçuk University, Konya, Türkiye

**Keywords:** Adhesives, Adhesive systems, Bond strength, Composite resins, Dental bonding, Dental restoration repair, Immediate dental restoration, Shear strength

## Abstract

**Background:**

Composite resin restorations frequently require repair due to fractures, marginal defects, or esthetic failures. The effectiveness of composite repair depends heavily on the surface treatment applied prior to bonding. This study aimed to evaluate the effects of various surface treatment protocols on the shear bond strength(SBS) between composite resin layers in same-visit immediate repair(IR) and delayed repair (DR) conditions, using bulk-fill and nanohybrid composite resins.

**Methods:**

Two types of composite resin materials—NeoSpectra ST HV (nanohybrid) and Tetric N-Ceram Bulk-Fill—were tested. A total of ten surface treatment protocols were evaluated under both immediate and delayed repair conditions (*n* = 10 per subgroup). Following grinding with a diamond bur, the surfaces were treated with one of the following: phosphoric acid, universal adhesives (Single Bond Universal, Prime&Bond Universal), three application modes of Clearfil SE Bond (two-step, bond only without primer, or phosphoric acid application followed by bond only), Optibond FL without primer, or GC Modeling Liquid. Additionally, direct layering without any surface treatment was included as a control. SBS was measured using a universal testing machine, and failure modes were analyzed using a stereomicroscope. One-way analysis of variance and Tukey’s post hoc test were used for statistical analysis (*p* = 0.05).

**Results:**

IR groups showed significantly higher SBS than DR groups across both composite types. The highest SBS were observed with GC Modeling Liquid and hydrophobic bonding agents, particularly in the bulk-fill immediate repair groups. Phosphoric acid application alone, or in combination with self-etch adhesive, did not improve bond strength. Universal adhesives did not outperform hydrophobic agents in any condition. Direct layering without treatment resulted in the lowest bond strength, especially in delayed repair.

**Conclusions:**

Surface treatment protocol significantly affects the success of composite resin repair. Hydrophobic adhesives and GC Modeling Liquid proved to be the most effective options, especially for same-visit immediate repairs. Simplified approaches avoiding unnecessary etching or priming may offer clinically efficient solutions without compromising bond strength.

## Background

Composite resin restorations are widely used in dentistry due to their conservative approach, aesthetic benefits, cost-effectiveness, and ease of repair [1]. A key factor in their success is the adhesion between resin layers, which is strongly influenced by the oxygen-inhibited layer (OIL) formed during polymerization. When resin is cured in the presence of oxygen, a superficial layer of unpolymerized monomers with a liquid-like consistency remains. During composite layering, the OIL increases the contact area between layers, enhances bonding, and facilitates the formation of an interdiffusion zone where copolymerization occurs, allowing for the development of a chemical bond between materials [2].

The clinical removal of the OIL, whether during immediate corrections or in the repair of aged restorations, directly affects the quality of adhesion between composite layers. In various cases, such as proximal contact problems, marginal incompatibility or excessive or incorrectly applied composite layers that cause problems in achieving colour and translucency harmony of the restoration, it may be necessary to cut back newly polymerised composite resin with a bur for corrections, during which the OIL is removed. This layer is also absent when old restorations need to be repaired. The OIL has been shown to enhance bond strength by enabling covalent interaction within the polymer network [3], raising concerns about the bonding quality when new composite is applied to surfaces lacking this layer [4]. Conversely, it has been suggested that the OIL may weaken the bond due to its fragile structure [5].

On the other hand, studies have reported that even after the removal of the OIL—which is rich in resin components—from freshly cured composite, free radicals remain within the material. The stability of these radicals is influenced by multiple factors. Although unreacted methacrylate groups persist in aged composites, the number of unsaturated double bonds decreases over time, reducing the bonding potential of newly applied composite. Consequently, the bond strength in immediate repair(IR) is generally higher than in the repair of aged composites [6]. For the repair of aged restorations, interventions such as surface roughening, adhesive application, or silane treatment are commonly recommended to enhance bonding between composite layers [7]. However, it remains uncertain whether such procedures are necessary to improve bonding in the repair of freshly polymerized composite restorations.

Given the uncertainty regarding the optimal repair approach for freshly polymerized versus aged composites, this study aims to assess the effects of various surface treatments on the shear bond strength (SBS) between composite resin layers following diamond bur roughening and the addition of a new composite layer. Comparisons will be made between freshly cured and aged composites, using two types of composite materials: bulk-fill and nanohybrid.

Null Hypotheses:

1. Surface treatments do not affect the SBS between layers of freshly polymerized composite resins after diamond bur roughening.
2. Surface treatments do not affect the SBS between layers of aged composite resins after diamond bur roughening.
3. There is no difference in the effect of surface treatments on SBS between freshly polymerized and aged composite resins.
4. There is no difference in the effect of surface treatments on SBS between bulk-fill and nanohybrid composite resins (NHC).

## Materials and methods

This study was approved by the ‘Selçuk University Faculty of Dentistry Non-Interventional Clinical Research Ethics Committee’ on December 9, 2022, with approval number 2022/55, and conducted at the Research Center Laboratory of the Faculty of Dentistry, Selçuk University.

### Experimental groups

**Fig. 1:**
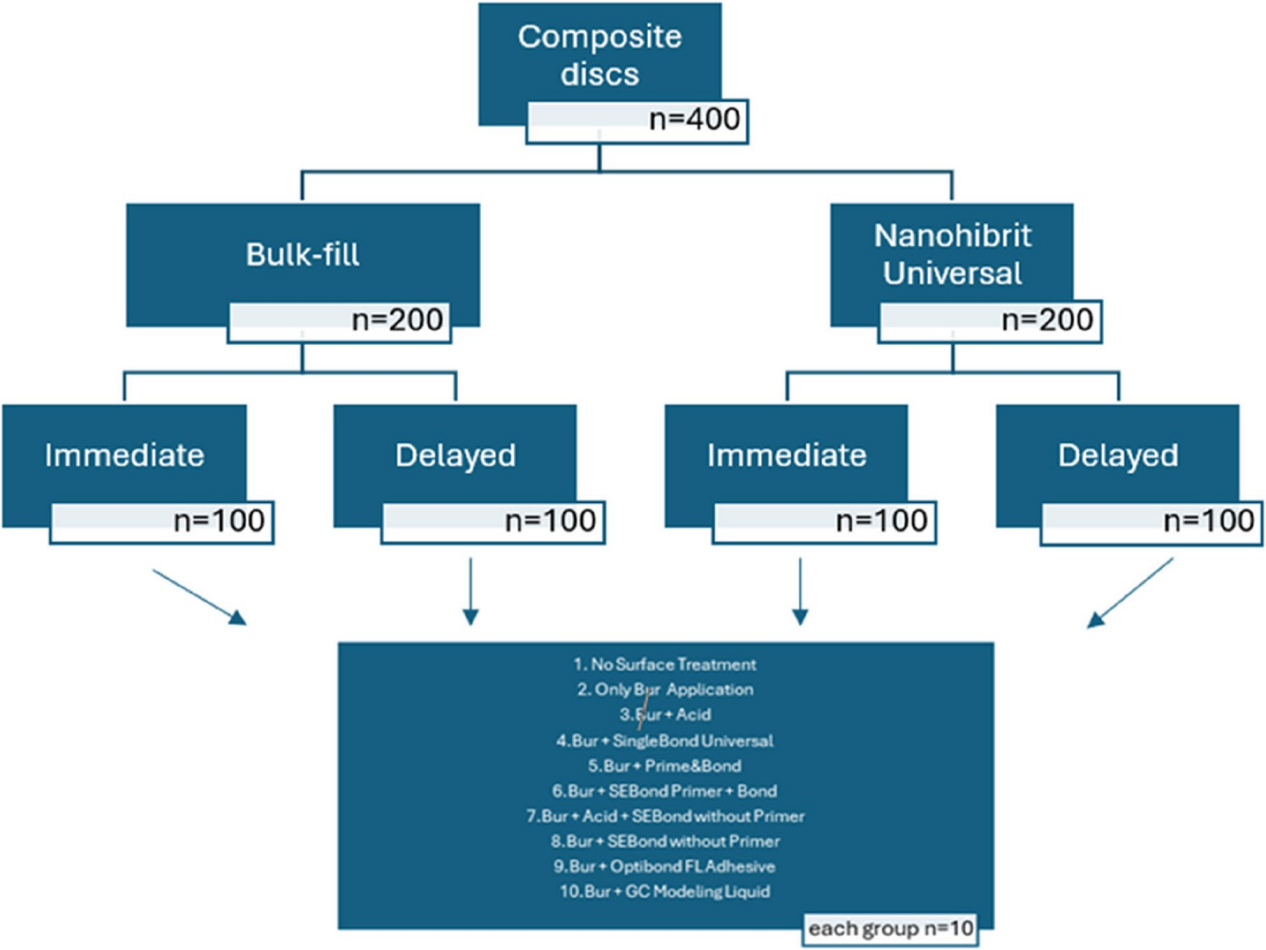
Study design and grouping of composite disc samples

Two composite resin types were used in this study: a NHC NeoSpectra ST HV(Dentsply, Konstanz, Germany) and a bulk-fill composite resin (BFC) Tetric N-Ceram Bulk-fill(Ivoclar-Vivadent, Schaan, Liechtenstein) (Table 1). A total of 400 specimens were prepared—200 for each composite resin type. Each composite resin type was divided into 20 groups(total 40 groups): 10 groups for IR and 10 for delayed repair(DR), based on surface treatments, with 10 specimens per group (Fig. 1).

**Table 1:** Composition of the composite resins used in the study

Composite Resin Material	Composition
Neo Spectra ST HV (Dentsply, Konstanz, Germany)	**Resin matrix**: Methacrylate-modified polysiloxane (organically modified ceramic) dimethacrylate resin, ethyl-4 (dimethylamino) benzoate, bis(4-methylphenyl) iodinium hexafluorophosphate, Bis-EMA, UDMA, TEGDMA, Camphorquinone **Fillers**: Spherical, pre-polymerized SphereTEC fillers (d3,50 ≈ 15 μm), unreacted barium glass, and ytterbium fluoride, Filler content by weight: 78–80%
Tetric N-Ceram Bulk Fill (Ivoclar-Vivadent, Shaan, Liechtenstein)	**Resin matrix**: Bis-GMA, Bis-EMA, UDMA, patented photo-initiator Ivocerin^®^ **Fillers**: Barium aluminum silicate glass, Ytterbium, Trifluoride, Filler content: approximately 55% (volumetric), 77% (by weight)

Except for the control groups that received no surface treatment(NST), all specimens were roughened using a yellow-band knife-edge diamond bur. Following this step, different surface treatment protocols were applied depending on the materials used. These included 37% phosphoric acid(Panora 200, Imicryl, Konya, Türkiye); two universal adhesives—Single Bond Universal(3 M ESPE, St. Paul, MN, USA) and Prime&Bond Universal(Dentsply/Caulk, Milford, DE, USA)—; a two-step self-etch adhesive, Clearfil SE Bond(Kuraray Dental, Tokyo, Japan); a three-step etch-and-rinse adhesive, OptiBond FL(Kerr, Orange, USA); and GC Modeling Liquid(GC Corp, Tokyo, Japan), which is a modeling resin designed to improve adaptation between layers (Table 2). Clearfil SE Bond was used in three forms: as a conventional two-step application, as primer-free, and as primer-free following phosphoric acid etching. In the “Bur + Acid + SEBond without Primer” protocol, after the diamond bur roughening, 37% phosphoric acid was applied to the surface for 15 s, followed by the application of adhesive resin.

**Table 2:** Composition and application procedure of adhesive Systems/Materials used in the study

Adhesive System/Material	Composition	Application Procedure
Single Bond Universal (3 M ESPE, St Paul, MN, USA)	10-MDP phosphate monomer, dimethacrylate resin, HEMA, Vitrebond™ copolymer, fillers, ethanol, water, silane, initiator	Applied by rubbing onto the surface for 20 s using an applicator. Gently air-dried for 5 s. Light-cured for 10 s.
Prime&Bond Universal (Dentsply/Caulk, Milford, DE, USA)	Bisphenol A-glycidyl methacrylate (Bis-GMA), urethane dimethacrylate (UDMA), triethylene glycol dimethacrylate(TEGDMA), phosphoric acid-modified acrylate resin(PENTA, MDP), initiator, stabilizers, isopropanol, water	Applied by rubbing for 20 s using an applicator. Gently air-dried for 5 s. Light-cured for 10 s.
Clearfil SE Bond (Kuraray Dental, Tokyo, Japan)	**Primer**: MDP, HEMA, hydrophilic dimethacrylate, camphorquinone, water. **Bond**: MDP, HEMA, Bis-GMA, hydrophobic dimethacrylate, N, N diethanol p-toluidine, camphorquinone, silanized colloidal silica	Primer applied by rubbing for 20 s. Gently air-dried for 5 s. Bond applied. Light-cured for 10 s.
Optibond FL (Kerr, Orange, USA)	**Primer**: HEMA, GPDM, PAMM, ethanol, water, photoinitiator. **Adhesive**: TEGDMA, UDMA, GPDM, HEMA, Bis-GMA, photoinitiator, fillers (SiO₂, barium aluminoborosilicate, Na₂SiF₆), bonding agent A174 (48% filler by weight)	37% phosphoric acid applied for 15 s, rinsed for 15 s, and air-dried for 5 s. Primer applied for 15 s, air-dried for 5 s. Adhesive applied. Light-cured for 10 s.
GC Modeling Liquid (GC Corp, Tokyo, Japan)	UDMA, 2-hydroxy-1,3 dimethacryloxypropane, 2-hydroxyethyl methacrylate, 2-hydroxy-1,3 dimethacryloxypropane triethylene glycol	Applied to the composite resin surface using a brush.
Panora 200 37% Etching Gel (Imicryl, Konya, Türkiye)	Phosphoric acid (37%)	Applied to the surface for 15 s, followed by rinsing for 15 s with water and air-dried for 5 s.
DIMEI Yellow-Banded Knife-Edge Diamond Bur (Dimei Medical Instruments Co., Shenzhen, China)	8.0/22.0 Dia: 1.0 mm	Used for roughening surfaces.

In the IR groups, repairs were carried out directly after specimen fabrication. In the DR groups, specimens were stored in distilled water at 37 °C for 15 days prior to repair. All specimens were then stored for an additional 24 h in distilled water at 37 °C before undergoing shear bond strength (SBS) testing.

### Specimen preparation

Composite disks(8 mm in diameter, 2 mm height) were fabricated using Teflon molds. Each disk was photopolymerized perpendicularly from the top surface for 20 s at 1000 mW/cm² using an LED curing unit (Valo, Ultradent Products, Inc., UT, USA) in standard power mode. Mylar strips were not employed during photopolymerization. After polymerization, the composite disks were embedded in autopolymerizing polymethyl methacrylate(PMMA)-based acrylic resin(Integra, Istanbul, Türkiye) within 1.5 cm diameter PVC pipe blocks, leaving the top surface exposed.

In the IR groups(excluding those without surface treatment), the samples were roughened unidirectionally for 20 s with yellow-banded knife-edge diamond burs immediately after polymerization. The samples were then washed with water for 5 s and air-dried for 5 s. The diamond bur used for every 5 samples was replaced with a new one.

In the DR groups(excluding those without surface treatment), the samples were incubated in distilled water at 37 °C for 15 days following polymerization, after which the same procedure used for the IR groups was applied.

Based on the surface treatments applied between the composite layers, 10 groups were created for both IR and DR of polymerized composite resins.

### Testing procedures

Following the surface preparation procedures applied in accordance with the manufacturer's instructions, a polypropylene mold (2.37 mm in diameter and 2 mm in height) was placed onto the surface of the composite samples, and a repair composite, identical to the composite resin used to form the disks, was applied.

**Fig. 2:**
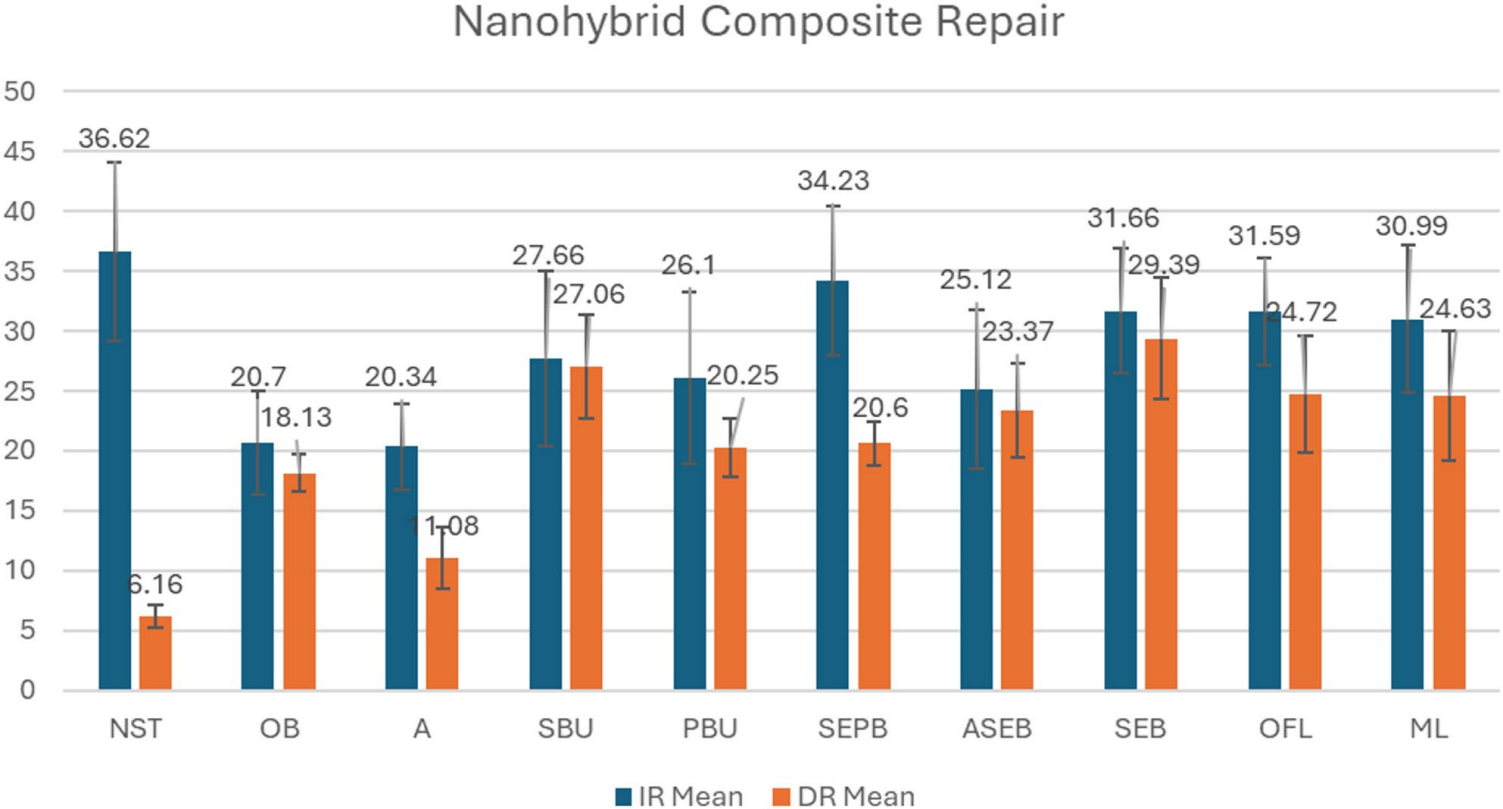
Mean shear bond strength (MPa) values of nanohybrid composite resin repair groups treated with different surface treatment protocols under immediate (IR) and delayed repair (DR) conditions

The samples were incubated in distilled water at 37 °C for 24 h. After this incubation period, they were subjected to an SBS test using a chisel-edge cutting blade with a loading rate of 1 mm/min on a universal testing machine (Instron, Marestek, Türkiye). The maximum load at failure was recorded in Newtons (N), and the SBS values (MPa) were calculated using the formula: S = F / A, where F is the fracture load (N) and A is the bonding area (mm²).

### Fracture type analysis

After the SBS test, the composite samples were examined under a stereomicroscope (Olympus, Tokyo, Japan) at 50x magnification. Fracture types were classified as follows: Adhesive Fracture: Fracture occurring at the interface between the composite resin and the adhesive surface. Cohesive Fracture: Fracture occurring within the material itself, either in the repaired composite resin or the tooth substrate. Mixed Fracture: Fracture involving both adhesive and cohesive failure simultaneously.

### Statistical analysis

In this in vitro study, the normality of the distribution of the samples was assessed using the Kolmogorov-Smirnov test. A one-way analysis of variance (ANOVA) was then employed to determine the differences in SBS values of composite repairs applied to NHC and BFC samples with 10 different surface treatments, both immediately and after a delayed period. Tukey’s HSD test was applied to identify the groups showing significant differences (*p* < 0.05). Statistical analyses were performed using SPSS Windows version 29.0.

## Results

### Shear bond strength test results

**Fig. 3:**
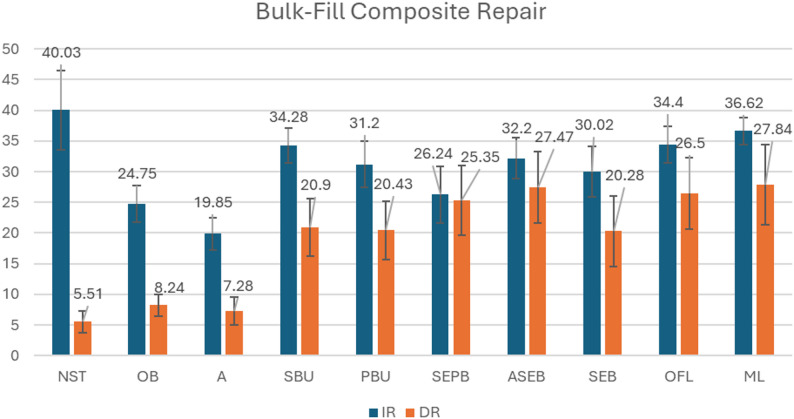
Mean shear bond strength (MPa) values of Bulk-Fill composite resin repair groups treated with different surface treatment protocols under IR and delayed repair (DR) conditions

In this study, the mean SBS values for the groups with different surface treatments applied to the specimens in IR and DR protocols are presented for NHC and BFC in Table 3; Figs. 2 and 3. The NHC IR NST group(Control) was used as the reference value for all NHC groups in both IR and DR, while the BFC IR NST group(Control) was accepted as the reference value for all BFC groups in both IR and DR. Overall, immediate repair (IR) resulted in higher shear bond strength (SBS) values than delayed repair (DR) for both NHC and BFC composites, and the effectiveness of surface treatments varied depending on the composite type and repair timing.

**Table 3:** The mean SBS values(MPa) and standard deviations for NHC and BFC in the IR and DR groups are presented. Values within the same column that share the same lowercase letters indicate no significant difference (Tukey’s test, *p* < 0.05), while values within the same row that share the same uppercase letters indicate no significant difference (Tukey’s test, *p* < 0.05)

Treatment Groups	NHC IR	BFC IR	NHC DR	BFC DR
No Surface Treatment(NST)	36.62 ± 7.47 (a, A)*	40.03 ± 6.42 (a, A)*	6.16 ± 0.95 (b, B)	5.51 ± 1.78 (b, B)
Only Bur(OB)	20.70 ± 4.31 (b, c,d, A)	24.75 ± 3.00 (b, e,f, g,A)	18.13 ± 1.58 (c, A)	8.24 ± 1.79 (b, B)
Acid(A)	20.34 ± 3.59 (b, c,d, A)	19.85 ± 2.57 (b, c,A)	11.08 ± 2.56 (b, c,B)	7.28 ± 2.26 (b, B)
SingleBond Universal(SBU)	27.66 ± 7.30 (c, d,e, f,g, A)	34.28 ± 2.79 (a, d,i, A)	27.06 ± 4.36 (d, f,h, A)	20.90 ± 4.64 (c, d,B)
Prime&Bond Universal(PBU)	26.10 ± 7.20 (b, c,d, e,f, A,B)	31.20 ± 3.71 (d, e,g, h,i, A)	20.25 ± 2.43 (e, f,B)	20.43 ± 4.71 (c, d,B)
SEBond P + B(SEPB)	34.23 ± 6.26 (a, g,A)	26.24 ± 4.61 (b, c,d, e,f, h,B)	20.60 ± 1.86 (e, f,B)	25.35 ± 5.66 (c, d,e, B)
Acid + SEBond without Primer(ASEB)	25.12 ± 6.66 (b, c,d, e,f, A,B)	32.20 ± 3.31 (a, d,g, h,i, A)	23.37 ± 3.95 (d, e,f, A,B)	27.47 ± 5.81 (d, e,f, A,B)
SEBond without Primer(SEB)	31.66 ± 5.22 (a, f,g, A)	30.02 ± 4.14 (d, e,g, h,i, A)	29.39 ± 5.03 (g, h,A)	20.28 ± 5.74 (c, d,B)
Optibond FL Adhesive(OFL)	31.59 ± 4.48 (a, f,g, A,B)	34.40 ± 2.97 (a, d,i, A)	24.72 ± 4.91 (d, e,f, h,B)	26.50 ± 5.84 (d, e,f, B)
GC Modeling Liquid(ML)	30.99 ± 6.13 (a, e,A, B)	36.62 ± 2.22 (a, i,A)	24.63 ± 5.39 (d, e,f, h,B)	27.84 ± 6.50 (d, e,f, B)

*In the control groups, the recommended layering technique was simulated without removing the OIL during composite restoration, and this was taken as the reference value for both repair time points

In the IR groups of NHC, SEPB, SEB, OFL, and ML demonstrated SBS values that were closest to the reference group, with no statistically significant differences. For BFC in the IR groups, ML, OFL, SBU, ASEB showed SBS values closest to the reference, without statistically significant differences. Additionally, these groups showed significantly higher SBS values than the Only Bur-treated specimens.

For NHC under DR conditions, all surface treatment protocols resulted in significantly lower SBS values compared to the reference, except for SEB, which showed no statistically significant difference. SEB yielded significantly higher SBS values than both SEPB and PBU groups. For BFC in the DR groups, all surface treatments resulted in significantly lower SBS values compared to the reference.

When comparing IR and DR groups of NHC, significant differences favoring IR were observed in the NST, A, and SEPB groups with identical surface treatments. In contrast, SBS remained consistent between IR and DR in the OB group, indicating minimal influence of repair timing.

When comparing IR and DR groups of BFC, significant differences favoring IR were observed in the NST, OB, A, SBU, PBU, SEB, and ML groups with identical surface treatments. Unlike NHC, a notable difference was observed in the Only Bur group, emphasizing the greater need for resin application in DR.

When comparing NHC and BFC with the same surface treatments under IR conditions, SEPB proved significantly more effective in NHC. When comparing DR groups, NHC showed significantly higher SBS than BFC in the OB group. This suggests that BFC may require additional adhesive or resin application after cut-back with bur in DR.

NST groups in IR, representing the cohesive strength of the composites, were used as reference values. SBS of the groups were calculated as percentages of these reference values (Table 4). None of the surface-treated groups reached the reference values, with ratios ranging from 13.8% to 93.4%.

**Table 4:** The percentage ratios of SBS data for NHC and BFC in IR and DR groups relative to their reference cohesive strength values

	NHC		BFC	
	IR	DR	IR	DR
NST	100%	16,8%	100%	13,8%
OB	56.5%	49.5%	61.9%	20.6%
A	55.5%	30.2%	49.6%	18.2%
SBU	75.5%	74.0%	85.7%	52.3%
PBU	71.2%	55.3%	78%	51%
SEPB	93.4%	56.2%	65.6%	63.3%
ASEPB	68.6%	63.8%	80.5%	68.6%
SEB	86.4%	80.2%	75%	50.7%
OFL	86.2%	67.5%	86%	66.2%
ML	84.6%	67.3%	91.3%	69.6%

### Fracture type analysis

The numerical distribution of fracture types obtained after all groups were subjected to SBS test is presented in Table 5.

**Table 5:** Numerical distribution of fracture types[Adhesive(A), Cohesive(C), Mixed(M)] based on composite resin type and repair timing

	Nanohybrid Universal Composite Resin						Bulk-Fill Composite Resin					
	Immediate Repair			Delayed Repair			Immediate Repair			Delayed Repair		
Fracture Type:	A	C	M	A	C	M	A	C	M	A	C	M
NST	0	10	0	10	0	0	3	5	2	9	1	0
OB	7	2	1	6	1	3	2	7	1	9	0	1
A	10	0	0	10	0	0	5	3	2	9	0	1
SBU	0	10	0	0	9	1	0	9	1	1	8	1
PBU	0	10	0	1	8	1	0	9	1	0	5	5
SEPB	0	10	0	0	8	2	0	10	0	0	8	2
ASEB	0	9	1	0	7	3	0	10	0	0	7	3
SEB	0	10	0	0	10	0	0	10	0	0	7	3
OFL	0	10	0	1	9	0	0	8	2	0	10	0
ML	0	9	1	1	8	1	0	7	3	0	10	0

Cohesive fractures were generally more frequent in groups exhibiting higher SBS values, whereas adhesive fractures were predominantly observed in the groups with lower bond strengths. In samples showing cohesive failure, the fracture typically occurred within the newly added repair composite layer.

The highest frequency of adhesive fracture type was observed in the NHC IR A group, NHC DR NST group, and NHC DR acid group. All samples in these groups exhibited an adhesive fracture pattern. The highest frequency of cohesive fracture type was observed in the following groups: NHC IR groups: NST, SBU, PBU, SEPB, SEB, OFL; NHC DR group: SEB; BFC IR groups: SEPB, ASEB, SEB; and BFC DR groups: OFL, ML. Cohesive fracture patterns were observed in all these groups. The highest frequency of mixed fracture type was observed in the BFC DR PBU group.

## Discussion

In this study, the effect of various interventions on the bond strength between composite resin layers during the addition of new composite resins onto freshly cured ones, following roughening with a diamond bur (IR), was evaluated in comparison to aged composite resins(DR) for two different types of composite resins. IR groups were designed to simulate re-layering procedures performed during the same clinical visit after correcting defects.

During the placement of direct composite resin restorations, clinical challenges such as contact issues, marginal discrepancies, or excess and improperly applied material affecting color and translucency may require the removal of newly polymerized composite using a bur. Diamond burs are therefore commonly used for practical and effective surface preparation in such repair procedures.

In contrast, the repair of aged restorations typically involves surface roughening and the application of bonding agents to enhance interlayer adhesion [7]. However, in the context of IR, residual carbon double bonds may still remain after OIL removal, enabling bonding with the newly added composite. To investigate whether bonding agents are equally essential in IR, our study applied various bonding resins between layers after roughening with bur in both BFC and NHC.

Diamond bur roughening was used in this study to simulate clinical conditions, as it has been shown to improve bond strength by creating macro- and micro-retentive features [8].

In IR protocols, the repair composite is typically matched to the substrate material; therefore, the same composite resin was used to standardize both IR and DR procedures in this study.

A 15-day water storage period at 37 °C was used as the aging protocol prior to repair. Rodrigues et al. [9] reported that composite specimens reached approximately 50% water saturation after nine days, and Biradar et al. [10] found that water absorption in composites peaks within the first week. Thus, the 15-day storage period was considered sufficient to achieve near-complete water saturation and to induce relevant changes in material properties. Moreover, Garcia et al. [11] demonstrated a marked decrease in free radical activity within 14 days after polymerization. Therefore, a 15-day aging time was selected to ensure reduced radical activity and to remain consistent with the literature.

Under ideal conditions, the SBS between composite resin layers is considered to approach the material’s cohesive strength. However, the direct bonding of new resin can be significantly reduced if the surface is contaminated, polished or aged [12]. To provide a baseline for comparison, specimens with intact OIL and no surface treatment—allowing immediate layering post-polymerization—were used as the control group for both IR and DR.

The cohesive strength values obtained from the NST groups under IR conditions were used as internal reference points to facilitate interpretation of the repair bond strength results and to enable standardized comparison across different surface treatment protocols.

Bonding effectiveness can be evaluated using both in vivo and in vitro methods; however, in vitro testing is preferred for its ability to standardize conditions and control variables [13]. In this study, the SBS test—one of the most widely used methods for evaluating repair bond strength—was chosen for its clinical relevance, especially in composite resin restorations. While adhesive test results allow for comparative evaluation of bonding systems [13], direct comparison of absolute values between studies should be avoided, as bond strength results may vary by 20–50% across similar studies [14].

Several studies have shown that composite-to-composite repair SBS can reach 20–80% of the initial SBS, considering the material’s cohesive strength [15] Similarly, in our study, IR groups exhibited SBS ratios of 49.6–93.4%, while DR groups showed ratios of 13.8–74%.

Studies have suggested that a bond strength between 15 and 25 MPa is appropriate for composite resin repairs, as it corresponds to typical dentin–composite bond strength values and is considered clinically acceptable [8, 16]. In this study, all IR groups achieved values within this clinically acceptable range. However, several DR groups fell below this threshold, including the NHC and BFC NST groups, NHC A group, and BFC OB and A groups. These results are consistent with previous findings and emphasize the importance of surface treatment in improving repair bond strength [17, 18].

The ideal layering technique involves applying fresh composite directly onto the OIL of previously polymerized material to optimize interlayer bonding. While bonding can occur even in the absence of OIL [19], residual free radicals and unreacted carbon–carbon double bonds play a key role in achieving this adhesion [20, 21]. These reactive groups remain most active within the first 24 h after polymerization [20]. Copolymerization with these groups enhances bond strength, whereas a high degree of conversion may reduce covalent interaction [19]. Atalay et al. [22] also confirmed that unreacted acrylate groups during IR improve the bond to new composite. In line with these findings, our study showed higher SBS values in IR for all surface treatments and composite types, likely due to greater availability of unreacted double bonds. The highest SBS was observed in untreated IR groups, attributed to the presence of the OIL.

In IR groups, acid etching after roughening with bur showed no additive effect. Specifically in BFC IR, acid application resulted in significantly lower SBS values compared to all resin-applied groups. Similarly, in NHC IR, applying primer before SEBond’s Bond led to better outcomes than acid application. In addition, universal adhesives (SBU and PBU) demonstrated inferior performance in NHC IR groups, with SBS values not reaching those of SEPB, SEB, OFL, or ML. Notably, the PBU group exhibited significantly lower bond strength than the SEPB group.

The null hypothesis stating that “Surface treatments do not affect the SBS between layers of freshly polymerized composite resins after diamond bur roughening.” was rejected. OFL and ML consistently produced bond strength values closest to the reference in both composite types. Moreover, these groups exhibited significantly higher SBS compared to specimens that underwent surface roughening with a bur alone, indicating their enhanced performance in immediate repair conditions. These findings suggest that OFL and ML may offer more clinically favorable outcomes, especially when the type of composite substrate is uncertain.

In the DR groups, NHC exhibited significantly higher SBS than BFC in the OB group, suggesting that BFC may require additional adhesive or resin application following bur treatment. This is consistent with Bonstein et al. [23], who reported that diamond bur roughening improves bond strength in aged composite repair, and with Atalay et al. [22], who found that bur roughening alone is insufficient for optimal bonding in bulk-fill composites. Our findings align with the results reported by Valente et al. [24], which demonstrated that combining diamond bur roughening, phosphoric acid etching, and adhesive application significantly improves long-term repair bond strength in aged composites. The strong interfacial bond achieved with this protocol may be explained by the macro-retentive features created by diamond bur roughening, improved surface wettability, and potential chemical interaction between the substrate and repair resin facilitated by the adhesive agent [25].

In both composite types, significant differences in SBS were observed among DR groups depending on the surface treatment applied. Particularly, NST and A groups consistently exhibited the lowest SBS values, while SEBond-containing groups, OFL, and ML provided superior outcomes. These findings indicate that surface treatment selection plays a critical role in optimizing repair performance for aged composite substrates. The null hypothesis stating that “surface treatments do not affect the SBS between layers of aged composite resins after diamond bur roughening” was rejected.

Composite resins are subject to hydrolytic degradation over time due to water diffusion, leading to matrix–filler interface weakening, increased porosity, and surface changes that can impair repair bond strength [26]. To simulate intraoral aging, specimens in this study were stored in distilled water for 15 days, a duration shown to reduce free radical activity and reactive methacrylate availability on the surface [11]. Consequently, surface treatments are necessary to re-establish macro-, micro-, or chemical bonding between aged and fresh composites [27]. In line with previous findings, DR groups in both composite types showed lower bond strength values than IR groups. The null hypothesis stating that “There is no difference in the effect of surface treatments on SBS between freshly polymerized and aged composite resins.” was rejected.

NHCs are widely used in both anterior and posterior restorations due to their favorable mechanical properties and esthetic advantages. Their nanofiller content may reduce the degree of conversion by increasing light scattering, potentially enhancing chemical bonding during IR by leaving more unreacted monomers available at the surface [28]. This could explain the superior performance of NHC compared to BFC in DR groups treated only with bur.

Compared to NHC, BFCs possess different polymerization characteristics that influence their repair behavior. BFCs, designed primarily for posterior use, allow for 4–5 mm increments, improving clinical efficiency. Their modified formulation promotes deeper polymerization and reduces polymerization shrinkage stress [29]. However, the high degree of conversion and more efficient photoinitiator systems in BFCs may have led to the significantly lower bond strength values observed in the OB group, where no additional resin was applied, and also in the SEPB group. This is likely due to the reduced availability of reactive methacrylate groups [30]. The null hypothesis stating that “there is no difference in the effect of surface treatments on SBS between bulk-fill and nanohybrid composite resins” was partially rejected.

Consistent with previous studies [23, 25], our results demonstrated that acid etching did not improve repair bond strength in DR and, in some cases, led to clinically unacceptable values. This observation aligns with reports indicating that acid treatment has minimal or no effect on bond strength [31]. In the NHC DR group, acid application following bur roughening resulted in a substantial reduction in bond strength. In both IR and DR of NHC, phosphoric acid application prior to SEBond without primer did not improve SBS and was even associated with a slight, though statistically insignificant, reduction. These findings support previous studies reporting that phosphoric acid provides no added benefit when used before self-etch adhesives, likely due to its limited etching capability or potential to damage the composite surface [22, 32].

The effectiveness of intermediate resin layers in composite repair is attributed to chemical bonding with the filler or matrix components and micromechanical retention achieved through monomer infiltration into surface irregularities. Proper adhesive application enhances surface wettability and facilitates chemical interaction between aged and newly applied composites [33].

Although universal adhesives containing functional monomers such as 10-MDP and silane offer simplified application and are widely used in clinical practice [34], they did not demonstrate superior bond strength compared to other adhesive systems in this study. SingleBond Universal, which includes pre-hydrolyzed silane, showed higher—but not statistically significant—SBS than Prime&Bond in DR of NHC. These results are consistent with previous studies suggesting that silane-containing universal adhesives do not significantly improve composite repair bonding [35, 36]. The effectiveness of universal adhesives varies considerably depending on substrate composition, consistent with previous findings [37].

In NHC IR groups, the highest SBS was achieved with the use of a primer prior to SEBond application. Consistent with our findings, Cavalcanti et al. [38] reported that two-step self-etch adhesives produced bond strengths comparable to the control in IR. However, in NHC DR and BFC IR, the use of a primer before bonding did not provide additional benefit, suggesting that an extra priming step may be unnecessary and time-consuming in these situations. Similarly, Rathke et al. [39] recommended limiting primer application in clinical repairs to cases involving dentin exposure.

Modeling liquids, also known as wetting or modeling agents, are used to reduce surface tension and improve the handling of composite resins by preventing the material from sticking to shaping instruments during placement [40]. In this study, GC Modeling Liquid, used as a repair material, demonstrated high SBS in both IR and DR-particularly in BFC- and performed similarly to the control in NHC IR. Its favorable performance may be attributed to the absence of hydrophilic acidic monomers, which reduces the risk of interfacial degradation between composite layers.

The use of a hydrophobic, unfilled resin during composite layering has been reported to improve mechanical properties by reducing voids and interfacial defects between layers [41]. In contrast, the use of hydrophilic lubricants has been associated with inferior mechanical performance and more pronounced discoloration [42], likely due to their higher monomer content and increased fluid absorption, which may alter the superficial composition of the composite. In the present study, layering with GC Modeling Liquid after cut-back yielded favorable results, supporting its clinical applicability in restorative procedures. Notably, ML achieved the highest SBS values in both IR and DR conditions of BFCs, further supporting its effectiveness as a repair material.

In both composite types and repair periods, the highest incidence of adhesive failure was observed in A and NST groups, which also showed the lowest SBS values. In contrast, cohesive failures were predominantly seen in groups where adhesive resins, particularly ML and SEBond (with or without primer), were applied, often corresponding to higher SBS. However, no direct correlation between failure mode and SBS was established, and the predominance of cohesive failures may have limited the interpretability of shear bond test results. In general, cohesive failures indicate that the interfacial bond exceeded the internal strength of the composite, which supports the higher SBS values observed in these groups.

None of the surface treatment groups achieved the SBS values observed in the control group, where the OIL was preserved. IR resulted in significantly higher SBS than DR for both composite types. The application of bonding agents after bur roughening improved SBS, particularly in DR, while unbonded DR groups yielded clinically unacceptable values. Modeling Liquid and hydrophobic resins were among the most effective agents, and additional acid or primer application before their use proved unnecessary. Silane-containing universal adhesives did not outperform hydrophobic resins. Notably, in the DR condition, diamond bur roughening alone resulted in higher bond strength in NHC compared to BFC.

This in vitro study has several limitations. Only two composite types and a limited selection of adhesive systems were evaluated, which may restrict generalizability. The aging protocol consisted solely of 15 days of water storage and did not include long-term degradation methods such as thermocycling or prolonged aging. In addition, no cyclic loading or mechanical fatigue simulation was applied, even though composite restorations are subjected to continuous occlusal forces in the oral environment. Future studies incorporating extended aging protocols, cyclic loading, contamination scenarios, and a broader range of composite and adhesive materials would provide a more comprehensive understanding of composite repair performance.

## Data Availability

The datasets used and analyzed during the current study are available from the corresponding author on reasonable request.

## Abbreviations

SBS: Shear Bond Strength
IR: Immediate Repair
DR: Delayed Repair
NHC: Nanohybrid Composite
BFC: Bulk-Fill Composite
ST: Surface Treatment
NST: No Surface Treatment
SBU: Single Bond Universal
PBU: Prime&Bond Universal
SEPB: Clearfil SE Bond (Two-step protocol)
ASEB: Clearfil SE Bond (Acid etch + bond only)
SEB: Clearfil SE Bond (Bond only, no primer)
OFL: OptiBond FL
ML: GC Modeling Liquid
PMMA: Polymethyl Methacrylate
OB: Only Bur (surface roughening with no adhesive)
OIL: Oxygen-Inhibited Layer
MDP-10: Methacryloyloxydecyl Dihydrogen Phosphate
LED: Light-Emitting Diode
PVC: Polyvinyl Chloride
SPSS: Statistical Package for the Social Sciences
